# The Impact of a History of Pre-maturity on Viral Respiratory Infections in Children Under 2 Years of Age: A Propensity Score-Matching Analysis of in-hospital Complications and Mortality

**DOI:** 10.3389/fped.2020.499013

**Published:** 2020-09-18

**Authors:** Jessie Zurita-Cruz, Alejandro Gutierrez-Gonzalez, Leticia Manuel-Apolinar, José Esteban Fernández-Gárate, María Luisa Arellano-Flores, Roberto Alejandro Correa Gonzalez, Guillermo Vázquez-Rosales, Patricia Pérez Vieyra, Rocio Sanchez-Armas, Nelly Cisneros-González

**Affiliations:** ^1^Facultad de Medicina Universidad Nacional de México, Hospital Infantil de México Federico Gómez, Unit of Research in Medical Nutrition, Pediatric Hospital “Centro Médico Nacional Siglo XXI”, Instituto Mexicano del Seguro Social, Mexico City, Mexico; ^2^Computer Research Center of Instituto Politecnico Nacional, Mexico City, Mexico; ^3^Endocrine Research Unit, Centro Médico Nacional, Instituto Mexicano del Seguro Social, Mexico City, Mexico; ^4^Epidemiological Surveillance Coordination, Instituto Mexicano del Seguro Social, Mexico City, Mexico; ^5^Endocrine Research Unit, Centro Médico Nacional, Instituto Mexicano del Seguro Social, Mexico City, Mexico; ^6^Health Research Coordination, Instituto Mexicano del Seguro Social, Mexico City, Mexico; ^7^Infectology Department, Pediatric Hospital “Centro Médico Nacional Siglo XXI”, Mexico City, Mexico; ^8^Unit of Inhalation Therapy, Pediatric Hospital, Instituto Mexicano del Seguro Social, Mexico City, Mexico; ^9^Unit of Research in Medical Nutrition, Pediatric Hospital “Centro Médico Nacional Siglo XXI”, Instituto Mexicano del Seguro Social, Mexico City, Mexico; ^10^Epidemiological Surveillance Coordination, Instituto Mexicano del Seguro Social, Mexico City, Mexico

**Keywords:** respiratory tract infection, respiratory syncytial virus, epidemiology, bronchiolitis, pre-maturity

## Abstract

**Introduction:** A history of pre-maturity may be a risk factor for complications in patients under 24 months of age hospitalized for viral respiratory infections (VRIs).

**Objective:** To identify the impact of a history of pre-maturity on in-hospital complications and mortality in patients under 24 months of age who were hospitalized for VRIs over a period of 5 years.

**Material and Methods:** This was a propensity score-matched study. The database was compiled by physicians, electronically validated by engineers, and analyzed by statisticians. Patients diagnosed with VRIs (based on International Classification of Diseases [ICD-10]) codes B974, J12, J120–J129X, J168, J17, J171, J178, J20, J203–J209, J21, J210, J211, J218, J219, J22X, and J189) from 2013 to 2017 were enrolled in the study. The subjects were classified into two groups according to the absence or presence of a history of pre-maturity (P070, P072, P073). Patients with congenital heart disease (CHD) (Q20–Q26) were excluded. Length of hospital stay, in-hospital complications, surgical procedures, and mortality were analyzed.

**Statistical Analysis:** Patients were matched according to age. For comparisons between groups, Student's *t*-tests and chi^2^ tests were applied. A logistic regression model was constructed to identify factors related to in-hospital complications and mortality.

**Results:** In total, 5,880 patients were eligible for inclusion in the analysis. The average patient age was 14.25 weeks. The presence of pre-maturity (coefficient = 1.16), male sex, bronchopulmonary dysplasia (BPD), in-hospital infectious complications (coefficient = 11.31), and invasive medical procedures (coefficient = 18.4) increased the number of days of hospitalization. Invasive medical procedures (OR = 6.13), a history of pre-maturity (OR = 2.54), and male sex (OR = 1.78) increased the risk for in-hospital complications. In-hospital infectious complications (OR = 84.2) and invasive medical procedures (OR = 58.4) were risk factors for mortality.

**Conclusions:** A history of pre-maturity increased the length of hospital stay and the rate of in-hospital complications but did not increase mortality in patients under 24 months of age hospitalized for VRIs.

## Introduction

Viral infections are common causes of respiratory tract disease in the outpatient settings of hospitals without isolation units. It is estimated that for some viruses, such as respiratory syncytial virus (RSV), 95% of children experience at least one infection before 2 years of age (1, 2). Influenza, metapneumovirus, parainfluenza virus, and coronavirus infections are also relatively common (3).

The manifestations of viral infections vary widely, with a clinical spectrum that includes mild infections, which can be treated on an outpatient basis, to more serious forms that require hospitalizations of variable duration (4). Inadequate feeding or fluid intake, a history of apnea, lethargy, moderate to severe respiratory distress, and low oxygen saturation in the ambient air suggest the need for hospitalization and, in severe cases, admission to a pediatric intensive care unit (PICU) (5–7).

In general, the etiological diagnosis is made on the basis of clinical data, and an age of <2 years indicates a strong suspicion of VRIs (8). The respiratory infections requiring hospitalization in children under 4 years of age are predominantly of a viral etiology; only 10% are due to rhinoviruses, and the remaining 90% are due to RSV or a combination of RSV and rhinoviruses or bocaviruses (9–11). Although specific therapies for these viruses are not yet available, viral identification can help reduce antibiotic use (12).

Patients with a history of pre-maturity have a higher risk of complications from viral respiratory infections (VRIs) due to a deterioration of lung development and an inadequate immune system response (13, 14). The condition of bronchopulmonary dysplasia (BPD), in which structural abnormalities persist in the lungs, is also a complication, resulting in greater complexity and vulnerability as well as a torpid evolution in VRIs (15–18).

In developing countries, an increased frequency of respiratory infections has been described in children under 5 years of age (19, 20). As there are no data on the impact of pre-maturity in this population, the objective of our study was to identify the impact of a history of pre-maturity on in-hospital complications and mortality due to VRIs in patients under 24 months of age who were treated at the Instituto Mexicano del Seguro Social (IMSS) over a period of 5 years.

## Materials and Methods

A cross-sectional study was conducted in pediatric patients who were 1–24 months of age and treated at the IMSS between 2013 and 2017 for severe acute respiratory infections. The data used in this study were obtained from the System of Information of Integral Health Care and the hospital records of the System of Medical Statistics (DataMart) of the IMSS and the databases of family medicine units. At the IMSS, medical facilities are classified according to three levels: 1st-level facilities perform preventive measures and treat acute and chronic pathologies without complications; 2nd-level facilities address complicated pathologies, surgical interventions, and treatments that require hospitalization; and 3rd-level facilities are equipped to treat individuals with complex and complicated diseases. In the IMSS database, records were available for each patient as well as those who received medical treatment in the 1st-, 2nd-, and 3rd-level care clinics. The diseases of interest were identified with International Classification of Diseases (ICD-10) codes.

The data in the database were compiled by physicians who attended and diagnosed patients visiting hospitals affiliated with the IMSS; the data were electronically validated by a team of engineers and strategically analyzed by statisticians affiliated with specialized centers at the regional and state levels.

For this study, the classification of respiratory tract infections was carried out based on ICD-10 codes. Mortality and hospitalization records of patients from 1 to 24 months of age from 2013 to 2017 were described using the following ICD-10 codes: *B974*, which includes RSV; and *J12*, which includes viral *(J120–J129X)* or non-specific *(J168, J17, J171, J178)* pneumonia, acute bronchitis *(J20, J203–J209)*, acute bronchiolitis *(J21, J210, J211, J218, J219)*, acute lower respiratory tract infection or other respiratory disorders *(J22X)*, and non-specific pneumonia *(J189)*.

In addition to the aforementioned codes that indicated a high suspicion of a VRI, the codes for complications such as non-viral pneumonia *(J13, J13X, J14, J15, J151, J152, J156, J158, J159, J16, J181, J182)*, respiratory failure *(J96, J960, J969)*, and non-specific respiratory disorder *(J988, J989)* were included in the diagnoses.

Patients with congenital heart disease and hemodynamic involvement *(Q20–Q26)* were excluded.

A history of pre-maturity was identified by relevant ICD-10 codes *(P070, P072, P073)*. The patients were subclassified into pre-mature and extremely pre-mature groups according to the history of weeks of gestational age (wGA). Pre-maturity was defined as 29–36 wGA at birth and extreme pre-maturity as <29 wGA at birth.

The presence of bronchopulmonary dysplasia (BPD) was identified by code *P271*.

The present study included patients who, according to the records, were first hospitalized at the IMSS for respiratory tract infections.

Among the included patients, sex, age, length of hospital stay, in-hospital complications, and the reason for discharge were analyzed; the presence of BPD was also examined.

In-hospital complications were defined as any event or condition that was detrimental to the patient's health, as caused by an unintentional injury and recorded by the medical and nursing staff during hospitalization. In-hospital complications were grouped and labeled as infectious, respiratory, and cardiovascular complications.

An invasive medical procedure was defined as deliberate access to the body through an incision or a percutaneous puncture, where instrumentation is used in addition to the puncture needle or instrumentation is conducted through a natural orifice. Invasive procedures were performed by trained professionals who used instruments, such as endoscopes, catheters, scalpels, scissors, devices, and tubes. Only invasive procedures related to VRI were included.

The primary outcomes were the mean length of hospital stay, in-hospital complications, and invasive medical procedures in children 1–24 months of age.

### Matching

To minimize the impact of bias related to age at hospitalization, patients with a history of pre-maturity were matched to patients without a history of pre-maturity using propensity scoring. The propensity scores were based on age at hospitalization. Nearest-neighbor matching was performed at a 1:1 ratio without replacement. The caliper was set at 0.01. The pymatch library for Python v3.7 was used.

### Statistical Analysis

Quantitative variables are presented as the mean and standard error. Qualitative variables are presented as proportions and frequencies. The patients were divided into two groups: individuals with and those without a history of pre-maturity. Two subanalyses were performed to compare (1) patients who had a history of extreme pre-maturity with those who had a history of pre-maturity and (2) patients who had a history of pre-maturity with those who did and did not have BPD.

A non-normal distribution was observed for the quantitative variables (age and length of hospital stay) using the Kolmogorov-Smirnov test; therefore, log-transformation was used for statistical analysis. Student's *t*-test and χ^2^ analyses were applied for inferences.

To identify factors related to the length of hospital stay, multiple linear regression was performed. To identify the factors related to in-hospital complications and mortality, a multiple logistic regression model was constructed.

A value of *p* < 0.05 was considered statistically significant.

STATA v.12.0 was used for the statistical analyses.

According to the Helsinki Declaration, the protocol was evaluated and approved by the National Research and Health Ethics Committee of IMSS with registry number R-2014-785-024. For this study, it was not necessary to request informed consent because it is a secondary analysis of a database obtained to record data from the hospitals affiliated with the IMSS.

## Results

### Total Hospitalized Patients

**Figure 1:**
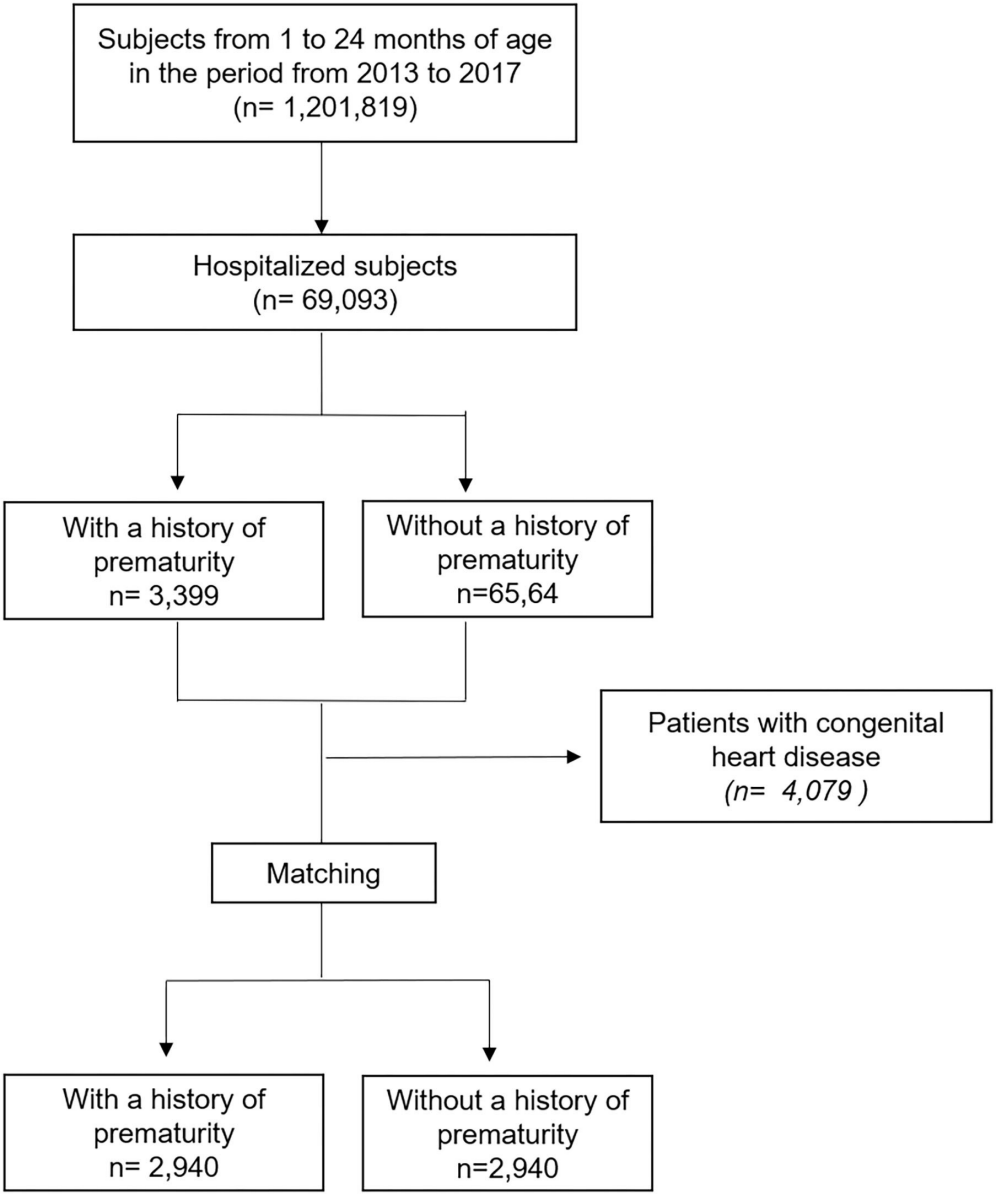
Flow chart.

There were a total of 69,093 hospitalizations due to VRI in children aged 1–24 months from 2013 to 2017 (Figure 1).

In the general population, a male predominance was observed (60.94%), with an average age of 14.17 weeks. A total of 826 patients (11%) had congenital heart disease, which had a higher prevalence in patients with a history of pre-maturity. During the hospital stay, a low frequency of complications was reported (1.79% *n* = 1,239), with the most frequent being bacterial coinfection of the respiratory tract (11.4% *n* = 141). Invasive medical procedures were performed for 603 patients (*n* = 603), and of these interventions, the most common was the placement of a venous catheter (40.6% *n* = 245), followed by endotracheal intubation (37.4% *n* = 226) and bronchoscopy with or without biopsy (21.9% *n* = 132). There was an increased frequency of invasive medical procedures in patients with a history of pre-maturity compared to patients with a history of full-term gestation (Table 1).

**Table 1:** Description of the characteristics of patients with a history of pre-maturity hospitalized for viral respiratory infections at the age of 1–24 months from 2013 to 2017 at the IMSS.

	**Total hospitalized patients**			**Matched hospitalized patients**		
	**Pre-maturity *n* = 3,399**	**Full-term *n* = 65,694**	***p***	**Pre-maturity *n* = 2,940**	**Full-term *n* = 2,940**	***p***
Age in weeks, mean (SE)	13.27 (0.24)	14.73 (0.06)	0.126	14.25 (0.26)	14.25 (0.26)	1.000
Sex: female (*n*, %)	1,298 (38.2)	25,686 (39.1)	0.288	1,106 (37.6)	1,302 (44.3)	0.001
Bronchopulmonary dysplasia (*n*, %)	455 (13.4)	0 (0)	0.001	349 (11.9)	0 (0)	0.001
Congenital heart disease (*n*, %)	459 (13.5)	3,620 (5.51)	0.001	-	-	-
Days of hospital stay mean (SE)	5.26 (0.10)	4.12 (0.17)	0.001	5.15 (0.11)	3.46 (0.04)	0.001
In-hospital complications (*n*, %)	122 (3.6)	1,117 (1.7)	0.001	88 (2.9)	27 (0.9)	0.001
Invasive medical procedures (*n*, %)	62 (1.8)	541 (0.8)	0.001	46 (1.5)	0 (0)	0.001
Death (*n*, %)	43 (1.3)	365 (0.5)	0.001	29 (0.9)	27 (0.9)	0.788

*SE, standard error*.

Among the hospitalized patients, there was a mortality rate of 0.59% (*n* = 408); the most frequent cause was respiratory failure secondary to pneumonia (*n* = 191) and heart failure.

Within the group of patients with a history of pre-maturity, 128 (1.9%) were identified as having a history of extreme pre-maturity. The age of the patients during hospitalization was 13.27 weeks, and there was a female predominance. In this group, the average length of hospitalization was 7.62 days; when the days of hospital stay were compared among patients with a history of extreme pre-maturity and pre-maturity, it was observed that patients with the former had a longer hospital stay (Table 2).

**Table 2:** Comparison of the characteristics of patients with a history of extreme pre-maturity and pre-maturity hospitalized for viral respiratory infections at the age of 1–24 months from 2013 to 2017 at the IMSS.

		**Extreme pre-maturity**	**Pre-maturity**	***p***
		***n* = 128**	***n* = 3,271**	
Age in weeks, mean (SE)		13.27 (1.09)	14.4 (0.25)	0.821
Sex: female (*n*, %)		54 (42.19)	1,244 (38.03)	0.342
Hospitalization year (*n*, %) 2013		24 (18.75)	852 (26.05)	0.070
	2014	27 (21.09)	609 (18.62)	
	2015	21 (16.41)	703 (21.49)	
	2016	29 (22.66)	518 (15.84)	
	2017	27 (21.09)	589 (18.01)	
Bronchopulmonary dysplasia (*n*, %)		19 (14.8)	436 (13.3)	0.622
Congenital heart disease (*n*, %)		17 (13.3)	442 (13.5)	0.940
Days of hospital stay, mean (SE)		7.62 (1.48)	5.17 (0.09)	<0.001
In-hospital complications (*n*, %)		5 (3.91)	117 (3.58)	0.884
Invasive medical procedures (*n*, %)		6 (4.69)	56 (1.71)	0.014
Death (*n*, %)		3 (2.34)	40 (1.22)	0.266

*SE, standard error*.

### Matched Hospitalized Patients

As shown in Table 1, there was no significant difference between the groups according to age at hospitalization; however, there was a difference in the proportion of male patients (Table 1).

Although the results suggested that patients with a history of pre-maturity had longer hospital stays during a VRI than those without a history of pre-maturity, the difference in the length of hospital stay between the groups increased when patients were matched. In addition, 1.96% of the patients (*n* = 115) experienced some type of in-hospital complication. Of these hospital complications, 64 were infectious, 24 were cardiovascular, and 28 were respiratory. With respect to invasive medical procedures, 46 patients underwent some type of procedure, including central venous catheter placement in 15 patients, bronchoscopy in 17 patients, and endotracheal intubation in 14 patients. Overall, the rates of in-hospital complications (infection in pre-maturity [1.26%] vs. full-term gestation [0.9%]; cardiovascular disease in pre-maturity [0.82%] vs. full-term gestation [0%], and respiratory illness in pre-maturity [0.95%] vs. full-term gestation [0%]) and invasive medical procedures (pre-maturity [1.5%] vs. full-term gestation [0%]) were higher in the group with a history of pre-maturity than in the group without a history of pre-maturity. However, there was no difference in the mortality rate between the groups.

Of the patients included with a history of pre-maturity (*n* = 2,940), a subanalysis was performed between those with (*n* = 349) and without (*n* = 2,591) BPD. Only a difference in invasive medical procedures (with BPD [4.01%] vs. without BPD [1.24%], *p* < 0.001) was observed, specifically in the placement of a central venous catheter (with BPD [2.29%] vs. without BPD [0.27%], *p* < 0.001) and in bronchoscopy (with BPD [1.43%] vs. without BPD [0.46], *p* = 0.042), which were higher in patients with BPD compared to those without BPD. No significant differences between the groups were found for the remaining characteristics, including age, sex, in-hospital complications, and death.

To identify factors related to the length of hospital stay, several multivariate models were built. We observed that the presence of pre-maturity (coefficient = 1.24), male sex, BPD, in-hospital infectious complications (coefficient = 11.28), and invasive medical procedures (coefficient = 18.42) increased the length of hospital stay (Table 3).

**Table 3:** Multiple linear regression analysis to identify the factors related to the days of hospital stay of children 1–24 months of age hospitalized for viral respiratory infections (*n* = 5,880).

	**Coefficient**	**Confidence interval 95%**	***p***
History of pre-maturity	1.16	0.93–1.38	<0.001
In-hospital infectious complications	11.31	10.27–12.35	<0.001
Invasive medical procedures	18.42	17.18–19.65	<0.001
Sex: male	0.65	0.43–0.86	<0.001
Bronchopulmonary dysplasia	0.76	0.33–1.19	<0.001

Regarding hospital complications, the multivariate model showed that invasive medical procedures (OR = 6.13), history of pre-maturity (OR = 2.54), and male sex (OR = 1.78) increased the risk for complications (Table 4A). Because respiratory and cardiovascular complications only occurred in patients with a history of pre-maturity, a multivariate model could not be performed. Nonetheless, in the multivariate model used to identify factors related to infectious complications, the only factor that was statistically significant was invasive medical procedures (OR: 11.32; 95% CI: 4.13–31.16; *p* < 0.001).

**Table 4:** Logistic regression analysis to identify the factors that impact on in-hospital complications (A) and mortality (B) of children hospitalized for viral respiratory infections (*n* = 5,880).

	**OR**	**Confidence interval 95%**	***p***
**A. In-hospital complications**			
Bronchopulmonary dysplasia	1.14	0.60–2.14	0.676
Sex: male	1.78	1.14–2.77	0.010
History of pre-maturity	2.54	1.62–4.00	<0.001
Invasive medical procedures	6.13	2.48–15.14	<0.001
**B. Mortality**			
Bronchopulmonary dysplasia	0.22	0.03–1.44	0.117
Sex: male	0.51	0.15–1.70	0.278
History of pre-maturity	1.85	0.60–5.62	0.277
Invasive medical procedures	58.4	3.31–541	<0.001
In-hospital infectious complications	84.2	51.6–542	<0.001

We also analyzed the proportion of deceased patients compared to living patients who had both in-hospital complications and invasive medical procedures, and a greater preponderance was observed among the former (10.7% of deceased patients vs. 1.29% of living patients, *p* < 0.001). To identify factors related to mortality, a multivariate model was constructed, showing that invasive medical procedures (OR = 58.4) and infectious complications were risk factors for mortality. In contrast, BPD, male sex, and pre-maturity had no significant association (Table 4B).

## Discussion

According to our observations, when matching patients according to age at hospitalization, it was possible to identify that patients with a history of pre-maturity had a higher frequency of in-hospital complications, particularly cardiovascular and respiratory complications, as well as procedures. Invasive VRI-related procedures were only observed in patients with a history of pre-maturity.

In developing countries, the pre-term birth rate is reported to be 762 per 10,000 births (21), which is higher than the corresponding rates reported in developed countries. The history of pre-maturity in infants with VRI can cause the clinical evolution of these patients to worsen the multiorgan immaturity that they present (22). Pre-mature birth interrupts the transfer of maternal antibodies in addition to the maturation of the immune system occurring at 6 months of life; thus, patients with a history of pre-maturity are susceptible to airway infections, particularly viral etiology (3). Patients with a history of pre-maturity may also experience failure in the continuous process of maturation of macrophages, which in turn can affect the functional responses of these cells and increase the susceptibility of an inadequate response and increased inflammatory process (4, 23). This may cause severe symptoms of respiratory infection and detachment of dead cells, which is aggravated by the relatively smaller airways than those in infants without a history of pre-maturity (24). Additionally, patients with a history of pre-maturity present structural changes in the lung, including increased bronchial muscle, collagen, and elastin (25). Furthermore, pre-mature exposure to high oxygen tension and growth restriction and other aspects of the extrauterine environment worsen these effects (24, 26). Our study revealed that patients with a history of pre-maturity have greater comorbidities, in addition to a greater severity of VRIs and therefore a longer hospital stay (27).

BPD is considered the most frequent chronic lung disease in infancy; it is estimated that 40% of pre-term infants between 22 and 29 wGA present with BPD (28), and in developing countries, the rate is 104 per 1,000 pre-term births younger than 32 wGA (29). Compared to healthy pre-term infants, these patients have been shown to have fewer mature macrophages in the airways in the immediate perinatal period. They also exhibit a decrease in HLA-DR expression in circulating monocytes (30). These conditions (both pre-maturity and BPD), render these patients more vulnerable to serious complications during VRIs. In our study group, 13.4% of the patients had BPD, which was a lower prevalence than that reported in previous studies (15–18).

As a risk factor for poor prognosis, treatment with invasive medical procedures is an indirect indicator of the disease severity in a patient. In this study, the most frequent procedures were bronchoscopy and the placement of a central venous catheter, which is an indicator of the requirement for long-term administration of medications.

A limitation of our study was that the data collected by ICD diagnoses did not include information about smoking or vaping at home, parental past or present history of asthma, the use of oxygen in the home, or breastfeeding. Additionally, the microbiological diagnosis of the respiratory infections was not performed, and the factors that influence the evolution of VRIs in these patients with BPD were not classified according to the severity of these diseases.

According to the results of this study, we recommend performing interventions, specifically hygienic measures and immunoprophylaxis, that prevent VRI in patients with a history of pre-maturity. The use of palivizumab prophylaxis is a crucial measure to reduce virus transmission and the frequent occurrence of VRI in patients under 2 years of age with a history of pre-maturity (31).

## Conclusions

A history of pre-maturity increased the length of hospital stay and in-hospital complications but was not related to an increased risk of mortality during hospitalization for VRIs in patients under 24 months of age who were treated at the IMSS over a period of 5 years.

## Data Availability Statement

The datasets analyzed in this article are not publicly available. Requests to access the datasets should be directed to Nelly Cisneros-González, zuritajn@gmail.com.

## Ethics Statement

The studies involving human participants were reviewed and approved by National Research and Health Ethics Committee of the IMSS (Registry Number R-2014-785-024). Written informed consent was not provided because for this study, it was not necessary to request informed consent because it is a secondary analysis of a database obtained to record data from the hospitals affiliated with the IMSS.

## Author Contributions

JZ-C analyzed and interpreted the data and wrote the article. LM-A and MA-F wrote the article. AG-G, JF-G, and RC analyzed and interpreted the data. PP and RS-A provided a critical review for important intellectual content, wrote the discussion, and approved the final version of the article. NC-G designed the study and acquired the information. All authors contributed to the article and approved the submitted version.

## Conflict of Interest

The authors declare that the research was conducted in the absence of any commercial or financial relationships that could be construed as a potential conflict of interest.
